# Getting the Hepatitis B Birth Dose Vaccine to Every Baby: A Rapid Scoping Review of Birth Dose Vaccine Delivery Strategies in Out-of-Facility Settings

**DOI:** 10.3390/vaccines14070554

**Published:** 2026-06-24

**Authors:** Sophia Knudson, Ankita Meghani, Katharine D. Shelley, Muluneh Yigzaw Mossie, Emily Grapa

**Affiliations:** 1 PATH, Seattle, WA 98103, USA; soph.knudson@gmail.com (S.K.); anki.meghani@gmail.com (A.M.); kshelley@path.org (K.D.S.); 2 PATH, Addis Ababa 1110, Ethiopia; yigzawm@gmail.com

**Keywords:** birth dose, vaccination, home births, newborn immunization, hepatitis B vaccines, scoping review, low- and middle-income countries, out-of-facility settings

## Abstract

Background/Objectives: Globally, coverage of the hepatitis B vaccine within 24 h of birth is 45 percent, far below the WHO target of 90 percent by 2030. For newborns delivered in out-of-facility settings, delayed contact with health workers, transportation barriers, and cold chain constraints can impede timely vaccination. This review explores strategies and facilitators for delivering birth dose vaccines to infants born outside of health facilities in low- and middle-income countries. Methods: A rapid scoping review was conducted, searching PubMed and targeted websites for peer-reviewed and gray literature published between 2005 and 2025. Data were charted using a standardized extraction tool. Frequency and thematic analyses were conducted. Results: After screening 315 studies, 26 eligible sources were identified. Delivery strategies consisted of three components: identifying and tracking home births; supporting caregiver uptake through education, reminders, or incentives; and delivering the vaccine through home-based administration or referral to facilities. Sub-components included pregnancy and birth notification systems, postnatal home visits, mobile reminders, incentives, and home-based vaccination by facility or community providers. The feasibility of these strategies was shaped by factors across system levels, such as national policies and financing; health system infrastructure; cold chain capacity; health workforce configuration; caregiver awareness; and community social norms. In several contexts, flexible cold chain approaches and vaccine administration by community-based cadres enabled timely vaccination of infants born at home. Conclusions: Vaccination programs can learn from existing out-of-facility vaccine delivery approaches to strengthen hepatitis B birth dose vaccination programs for timely and equitable coverage.

## 1. Introduction

Hepatitis B infection remains a major global public health challenge, with an estimated 254 million people living with chronic hepatitis B in 2022 and approximately 1.2 million new infections annually [1]. Mother-to-child transmission is a significant contributor to this burden, particularly in regions with high endemicity. However, administration of the hepatitis B birth dose vaccine to newborns within 24 h of birth, as recommended by the World Health Organization (WHO) [2], can prevent 75 to 95 percent of vertical transmissions from mother to child [3]. Despite its effectiveness, global timely birth dose coverage (within 24 h) remains at only 45 percent [4], far below the WHO’s target of 90 percent by 2030 [5].

A challenge to timely hepatitis B birth dose administration is the large proportion of births that occur in out-of-facility settings. Globally, 28 percent of births take place at home, with rates reaching as high as 78 percent in some countries [6]. Newborns delivered at home face an elevated risk of missing timely hepatitis B birth dose for two primary reasons. First, facility-based delivery and immunization strategies inherently exclude infants who are not born in, or do not immediately present to, health facilities, which creates systematic gaps in vaccine reach [7]. Second, logistical and health system constraints such as limited transportation options, long travel distances, shortages of trained vaccinators within communities, and cold chain limitations often impede rapid postnatal contact with health workers [8,9,10,11,12]. Given that the effectiveness of the hepatitis B birth dose declines when administration occurs beyond 24 h, these delays further compromise efforts to prevent vertical transmission.

Hepatitis B birth dose adoption and timely coverage vary by region. The WHO Western Pacific Region, where incorporation of the hepatitis B birth dose into national immunization schedules is relatively widespread [13], has achieved timely coverage of 79 percent [14]. In contrast, only 16 of the 47 countries in the Africa Region have introduced the hepatitis B birth dose into their routine immunization programs [13], corresponding to the region’s substantially lower timely coverage of 17 percent [14]. The slower pace of adoption, combined with the high proportion of out-of-facility births in many African settings [13,14], highlights the need for delivery approaches that extend beyond facility-based platforms. As more countries prepare to introduce the hepatitis B birth dose or strengthen existing programs, evidence on models designed specifically to reach babies born in out-of-facility settings is increasingly important.

To our knowledge there are no existing reviews that examine vaccine birth dose delivery strategies specifically designed to reach babies born outside of health facilities. To address this gap, we conducted a rapid scoping review in 2025 as the first phase of a mixed-methods hepatitis B birth dose learning initiative funded by Gavi, the Vaccine Alliance, in partnership with Unitaid [15]. The broader initiative assessed the feasibility, acceptability, cost, market access, and impact of innovative strategies for improving timely hepatitis B birth dose delivery to children born outside of health facilities in low- and middle-income countries (LMICs) [16].

A scoping review was selected to capture the breadth of delivery strategies, including both established and emerging models, implemented in LMICs. In the context of the Gavi-funded learning initiative, this scoping review was intended to rapidly assess the existing evidence to inform the subsequent human-centered design workshops aimed at identifying sustainable, context-appropriate delivery models [16]. The findings of this review can be used to support countries planning hepatitis B birth dose introduction or seeking to improve timely coverage, particularly among newborns most likely to be missed by facility-based approaches.

This rapid scoping review was guided by the following primary research questions:

1. What strategies have been used to deliver birth dose vaccines to newborns delivered outside of health facilities in LMICs?
2. What factors influenced the implementation of out-of-facility vaccination strategies?

In addition, this rapid scoping review included two secondary research questions to understand aspects of the cold chain and health workforce required to support vaccine delivery strategies:

1. What cold chain approaches, including outside the cold chain or controlled temperature chain (CTC), have been used to enable out-of-facility birth dose delivery strategies in LMICs?
2. What type(s) of health workers, including non-physician and community cadres (inclusive of community health workers [CHWs]), have implemented the out-of-facility birth dose delivery strategies (including vaccine administration) in LMICs?

## 2. Materials and Methods

This rapid scoping review was conducted following the Joanna Briggs Institute methodology for scoping reviews and is reported in accordance with the PRISMA extension for Scoping Reviews (PRISMA-ScR) checklist (provided in Supplementary Material Table S1) [17]. A review protocol was developed a priori to guide the methodology; however, it was not registered in a public repository due to the timeline and scope of the hepatitis B birth dose learning initiative within which this review was conducted [15]. The review was initiated as a rapid foundational evidence mapping to inform subsequent human-centered design activities. The protocol is included as Supplementary Material (S2). The rapid nature of this review influenced the methodological design, including the search strategy, screening process, and thematic coding, as described below [18].

### 2.1. Definitions

Throughout this review, the term “out-of-facility births” is used as the primary term to refer to births occurring outside of formal health facilities, including homes, community spaces, or other non-facility settings. Where original sources use alternative terminology (e.g., “home births”, “community births”, “births outside health facilities”), the original language is preserved in the description of that source, but “out-of-facility” is used in synthesized text throughout.

Additionally, two cold chain storage approaches referenced throughout this review are defined here for clarity. Outside the cold chain (OCC) refers to the transport and storage of vaccine vials outside of standard cold chain equipment for a defined period. Controlled temperature chain (CTC) is a WHO-defined approach that permits vaccines to be kept at ambient temperatures of up to 40 °C for a minimum of three days during the final stage of distribution [19]. Vaccines used under CTC must be specifically approved through national regulatory authorities and WHO prequalification. To date, no hepatitis B vaccine has received CTC approval. However, evidence of the vaccine’s relative heat stability [20,21,22] has led some studies included in this review to use off-label outside the cold chain approaches to enable home-based and community-based birth dose delivery while acknowledging this falls outside current regulatory approvals.

### 2.2. Eligibility Criteria

Eligibility criteria were defined using the Population, Concept, and Context framework [23], supplemented by additional parameters relevant to scope and feasibility. Table 1 presents the full inclusion and exclusion criteria.

### 2.3. Information Sources and Search Strategy

We conducted a comprehensive search of peer-reviewed research in PubMed using terms aligned with the population, concept, and context (full search strategy provided in Supplementary Material S3). Given that this was a rapid review, we prioritized one database. PubMed was selected as the primary bibliographic database given its strong coverage of global health, immunization, and LMIC-focused implementation science research, and because it is one of few databases recommended for evidence synthesis that does not require a subscription. Other commonly used databases for systematic and scoping reviews, including EMBASE, Scopus, Web of Science, and CINAHL, are subscription-based and were not available within the scope of this rapid learning initiative [24]. Google Scholar, while broad in coverage, was not used as a principal search system as it has been shown to fail key reproducibility and Boolean functionality requirements for systematic searches [24]. To capture insights from non-peer-reviewed materials (e.g., program reports, policy documents, conference presentations), we supplemented this with targeted website searches of relevant organizational and immunization resource platforms, including Gavi, the Vaccine Alliance; the WHO; the Coalition for Global Hepatitis Elimination; Technet-21; and the Boost Community. We used site-specific search filters where available and manual browsing of resource libraries to identify materials related to hepatitis B, routine immunization, newborn vaccines, and delivery strategies for out-of-facility births. A summary of all sources reviewed through targeted website searches, including document types and inclusion decisions, is provided in Supplementary Material Table S4.

Gray literature, including evaluation reports, country policies, and documents shared by immunization experts and project collaborators, was also included. All records were imported into Covidence for management and screening [25].

### 2.4. Literature Screening and Selection

Two independent reviewers screened 20 percent of the titles and abstracts for relevance based on the eligibility criteria. Agreement between reviewers was high (≥80 percent), after which screening proceeded with a single reviewer per source [26]. Full-text review was conducted for all eligible sources by one reviewer. Single-reviewer screening and review was adopted after the calibration phase due to the resource and time constraints inherent to the learning initiative. To mitigate potential selection bias, weekly meetings were held between both reviewers throughout the screening and full-text review phase to discuss borderline cases, resolve uncertainties, and maintain consistency in the application of eligibility criteria.

### 2.5. Data Charting and Data Items

Data extraction was performed using a standardized template developed within Covidence. The form was informed by the review’s research questions and components of the Population, Intervention, Comparison, and Outcome (PICO) Framework as practical organizational labels to structure the extraction fields [27]. The “Intervention” component of PICO was used to clearly define and capture the delivery strategy described in each source, given that the primary aim of this review was to characterize how birth dose vaccination strategies are operationalized in out-of-facility settings. Any results the source itself reported were captured under the “Outcome” field; however, the review did not assess the effectiveness of outcomes, as this fell outside the scope of a rapid scoping review. The data extraction form was pilot-tested on one study and refined before full application. Data charting included both close-ended categorical data elements and open-ended text fields to capture descriptive information. The data extraction form and definitions for all data elements are included in Supplementary Material Table S5.

### 2.6. Summarizing and Reporting Results

Frequency analyses were conducted for categorical data elements to describe characteristics of the included studies. Thematic analyses were conducted for open-ended data elements to identify categories and subcategories of delivery strategies, as well as barriers and enablers to their implementation. Barriers and enablers associated with the delivery strategies were synthesized using thematic coding and categorized across national, health system (organizational), and community levels. A hybrid inductive/deductive approach was used, with the initial coding structure informed by the data extraction domains and additional codes identified iteratively. Two reviewers were involved in coding, with each responsible for coding different domains of the data extraction template due to the rapid nature of this review. To promote consistency, the reviewers met regularly to discuss data interpretation, align on emerging codes, and resolve any disagreements. Consistent with scoping review methodology, no formal quality appraisal or risk-of-bias assessment was conducted. The included sources span a range of study designs and document types which vary in rigor. Where studies reported specific outcomes, these were described narratively; however, no comparative effectiveness conclusions are drawn. Reporting was guided by PRISMA-ScR [17].

## 3. Results

**Figure 1:**
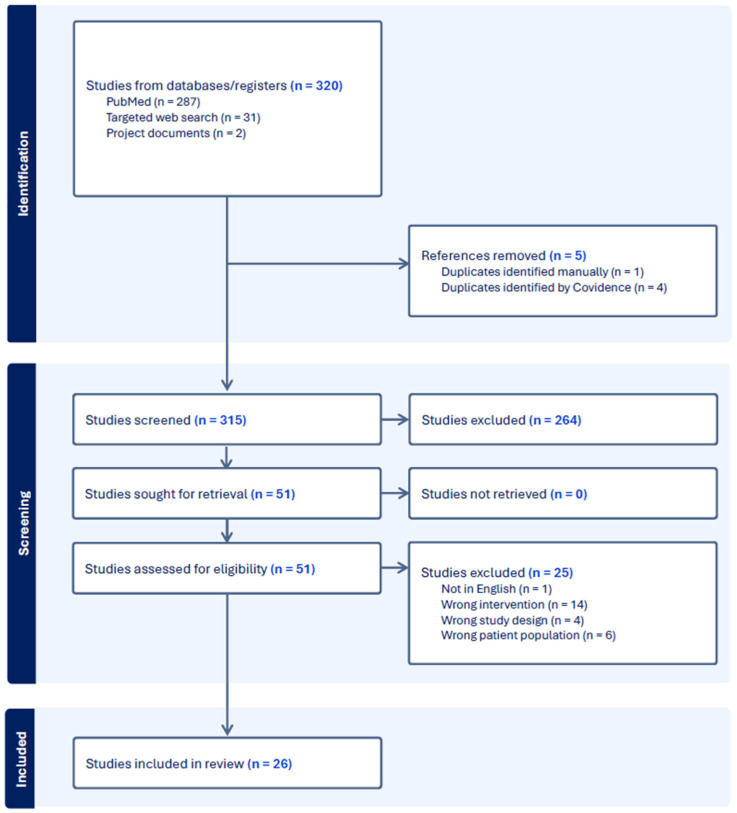
PRISMA flow diagram of the literature search and selection process.

The literature search yielded a total of 320 studies, comprising 287 articles from the PubMed database search, 31 studies from targeted website searches, and 2 unpublished country program reports shared by project collaborators. After removing 5 duplicates, 315 records were screened by title and abstract, resulting in 264 exclusions. The remaining 51 full-text articles were assessed for eligibility, of which 25 were excluded for reasons such as not addressing out-of-facility births, lacking a defined birth dose strategy, or focusing exclusively on facility-based birth dose delivery. A total of 26 records were included in the final review. The PRISMA chart in Figure 1 outlines the search results.

### 3.1. Characteristics of Included Studies

The 26 studies were published between 1 January 2005 and 1 April 2025 and represented the Africa Region (*n* = 10), Western Pacific Region (*n* = 9), South-East Asia (*n* = 1), and global contexts (*n* = 6). Most studies examined hepatitis B birth dose (*n* = 20), while others addressed BCG (*n* = 2) and OPV0 (*n* = 1), or a combined birth dose vaccine approach (*n* = 3). Target populations included children (*n* = 9), caregivers (*n* = 7), and health care providers (*n* = 7), while 5 systematic reviews and meta-analyses encompassed multiple population groups. Seventeen studies reported on health worker roles in vaccination efforts, and twelve described cold chain or packaging approaches.

The majority of included studies were peer-reviewed research articles (*n* = 19). The remainder were non-peer-reviewed program reports (*n* = 2), policy document (*n* = 1), and additional gray literature sources (1 training package, 1 Gavi application, and 2 conference presentations). Table 2 presents detailed characteristics for each study in this review.

### 3.2. Birth Dose Delivery Strategies

To address the first research question, what strategies have been used to deliver birth dose vaccines to newborns delivered in out-of-facility settings in LMICs, the included studies described a broad range of strategies implemented across diverse contexts. These strategies consisted of three components, reflecting both the enabling systems required to support out-of-facility vaccination and the approaches used to deliver the vaccine itself. First, the home births must be identified. Second, a strategy must be selected for administering vaccines to these newborns once they have been identified. Vaccine administration strategies fell into two categories: (a) strategies for vaccinating newborns at home or in the community and (b) strategies for encouraging mothers/caregivers to bring their newborns to a facility for vaccination. Third, there are interventions that can be implemented to encourage birth dose vaccination by improving awareness or education or providing incentives. Table 3 summarizes the components of a birth dose delivery strategy as identified in our review.

### 3.3. Enablers and Barriers to Implementing Out-of-Facility Strategies

To explore the conditions shaping implementation, the included studies reported a range of enablers and barriers influencing out-of-facility strategies (research question 2).

The enablers and barriers (Table 4) are grouped by their applicability to national level, health system level, or community level. Many of these factors can act as either enablers or barriers, depending on whether they are adequately planned for, supported, and resourced. While some studies identified these factors as enablers, others found them to be barriers. Importantly, these factors are relevant across a range of out-of-facility birth dose vaccine delivery strategies.

### 3.4. Cold Chain and Packaging Approaches

A secondary objective of this review was to understand the cold chain approaches, including outside the cold chain or controlled temperature chain, that have been used to enable out-of-facility birth dose delivery strategies in LMICs. Twelve studies described strategies related to cold chain infrastructure and management for out-of-facility birth dose delivery strategies. These studies described a range of modalities, including active cold chain using fixed-site refrigeration at health facilities (n = 5) [33,44,46,49,53], passive cold chain using vaccine carriers with conditioned ice packs during outreach or home visits (n = 5) [33,44,46,49,51], and packaging formats such as compact prefilled auto-disable devices used by both facility- and community-based vaccinators (n = 2) [44,47]. Several studies also described storage strategies that permitted flexibility beyond the traditional cold chain range, including outside the cold chain storage for monovalent hepatitis B birth dose vaccines for up to 28 days outside refrigeration (n = 6) [29,30,35,38,40,47], and controlled temperature chain approaches under which vaccines could be kept above 2–8 °C for a limited time prior to administration (n = 2) [44,49].

Among these studies describing flexible cold chain conditions, several reported the use of outside the cold chain approaches within the context of hepatitis B birth dose delivery outside of health facilities. In these studies, the hepatitis B birth dose was stored at peripheral facilities without cold chain equipment or directly with vaccinators, and was used during postnatal care home visits, outreach, or community-based follow-up of home births [30,47,49,53]. No studies described implementation of a controlled temperature chain approach for hepatitis B birth dose; of the two studies that mentioned controlled temperature chain, one is a WHO guidance document [43] and the other is a modeling analysis that examined scenarios in which the hepatitis B birth dose could be administered using a combination of approaches including controlled temperature chain with compact prefilled auto-disable devices [44]. Studies that described compact prefilled auto-disable device usage were used in combination with outside the cold chain or controlled temperature chain approaches and allowed for easier administration outside of health facilities and by cadres beyond facility-based staff [29,31,44,45].

### 3.5. Health Worker Roles

Finally, we examined the cadres of health workers, including non-physician and community cadres, that have implemented out-of-facility birth dose delivery strategies in LMICs. Across the 17 studies that reported on cadres involved in out-of-facility strategies, a range of providers contributed to the identification, referral, and vaccination of home-born infants. In some settings, facility-based nurses or doctors administered birth dose vaccines (n = 8) [33,36,41,43,44,46,49,53], requiring caregivers to bring newborns to facilities shortly after home birth. Other studies described vaccination by CHWs (n = 4) [44,47,49,51], midwives and traditional birth attendants (n = 6) [29,35,46,49,51,52], village doctors or village-level health workers (n = 2) [35,36] who were trained to administer the vaccine during home visits, outreach, or community follow-up.

Several of the studies highlight the supportive roles of CHWs and other community cadres, including identifying and tracking home births, notifying health facilities, and referring caregivers as well as educating them about the importance of timely hepatitis B birth dose vaccination [40,41,46,48]. In most of the studies, CHWs are described as not administering the vaccine themselves but playing a critical role in facilitating access to vaccination services through maintenance of pregnancy registers and sharing them with facilities, notifying health workers of imminent or recent home births, and engaging with the community through culturally appropriate education and communication activities. An exception was noted in a study by Wang et al. (2007), in which village health workers in China had been trained to administer the hepatitis B birth dose at home. This strategy, combined with outside the cold chain storage of vaccines in village clinics or homes, substantially improved timely coverage by overcoming barriers such as transportation and cultural postpartum isolation practices [47].

## 4. Discussion

This rapid scoping review provides a consolidated overview of delivery strategies designed to reach newborns delivered in out-of-facility settings with birth dose vaccines in LMICs. By mapping evidence from 26 sources across multiple regions and birth dose vaccine types, we observed that out-of-facility delivery strategies consistently organize around three functional components: (1) identifying home births, (2) supporting caregiver uptake through education or incentives, and (3) providing a mechanism for delivering the vaccine—either at home or by referral to a facility. These components align closely with the WHO global guidance on hepatitis B birth dose vaccination, which outlines key functions needed to reach newborns born at home, such as timely birth notification, engaging families and communities, and making vaccination feasible either through home-based administration or by supporting caregivers to reach a facility [43]. Our component structure reflects these same functions but organizes them in a way that highlights the common steps across out-of-facility birth dose delivery strategies, regardless of the birth dose vaccine administered. Building on these foundations, this review offers a menu of implementation options within each component that countries can consider as they plan for introduction and scale-up of hepatitis B birth dose. This review documents a spectrum of strategies implemented under different conditions, reflecting the wide variability in health system structures, community norms, and resource environments. The goal is therefore not to prescribe, but to equip countries with a clear landscape of what has been tried, how strategies were operationalized, and what contextual factors shaped implementation.

A second key takeaway from this review is that system-level enablers and barriers significantly influence the operationalization of these strategies. A successful out-of-facility birth dose vaccination strategy depends on the alignment of national policy commitment, health system readiness, and community dynamics. At the national level, strong political support, partner collaboration, clear operational policies, and coordinated advocacy help create the conditions for implementation, while inconsistent guidance or external disruptions (e.g., COVID-19) can hinder delivery. We categorized most determinants at the health system level, where consistent service availability, strong linkages between facility and community levels of the health system, reliable supply chains, confident and well-trained providers, and active coordination between immunization and maternal, newborn, and child health (MNCH) programs influence whether newborns receive a timely birth dose. Community level factors interact with and often amplify health system dynamics. Caregiver knowledge, cultural expectations regarding newborn care at home, and local perceptions of vaccine safety influence whether families are prepared for timely vaccination and whether outreach strategies can be effectively implemented. The national, health system, and community levels at which these determinants operate are consistent with how implementation science frameworks, such as the Consolidated Framework for Implementation Research (CFIR) [55,56], conceptualize the multi-level nature of implementation context.

Notably, the same factor often functioned as an enabler in one setting and a barrier in another. For example, in Madagascar, low awareness among caregivers of birth dose vaccine recommendations was identified as a key constraint [28], whereas Moturi et al. found that hepatitis B birth dose vaccination was highly acceptable and there were no observed caregiver refusals based on knowledge, attitudes, and practice assessments in five African countries [35]. One can imagine that differences in context may influence whether a factor is an enabler or barrier. For instance, selective vaccination policies based on maternal screening may be efficient where laboratory capacity is strong, but in settings without reliable screening systems they can delay vaccination and increase missed opportunities. This type of context dependence and influence of interrelated barriers and enablers across system levels reflects the conclusions from a 2023 scoping review of hepatitis B birth dose vaccination programs in Africa in which Solomon-Rakiep et al. emphasize the importance of taking a system view and recognizing the complexity of hepatitis B birth dose vaccination [8]. This underscores the need for countries with high rates of home births to consider system readiness and contextual fit as they explore out-of-facility delivery options. Drawing on the enablers and barriers documented in this review, such an assessment should consider at minimum: whether national policy and operational guidance for out-of-facility birth dose vaccination are in place or in development; the capacity and reliability of supply chain and cold chain systems; the design and scope of practice of existing community health worker programs, including whether vaccination task-shifting is operationally feasible; the strength of coordination between immunization and MNCH programs at facility and community levels; existing levels of caregiver awareness and community acceptability of home-based vaccination; and the availability of sustainable financing for outreach-based delivery models. These readiness dimensions map directly onto the six building blocks of the WHO Health Systems Framework (service delivery, health workforce, access to essential medicines and technologies, financing, health information systems, and leadership and governance) [57], reinforcing that strengthening out-of-facility birth dose delivery requires coordinated action across the health system. Countries need not have all these conditions fully in place before proceeding, but understanding where gaps exist will help determine which strategies are most feasible and what enabling investments may be needed alongside implementation.

Across the reviewed studies, flexible cold chain strategies emerged as a promising approach to increase timely hepatitis B birth dose vaccination among newborns born in out-of-facility settings. Outside the cold chain approaches demonstrated feasibility and strong potential to overcome infrastructure challenges in settings lacking reliable electricity or refrigeration, enabling vaccinators to reach infants during home visits and community follow-up. Yet, despite repeated successful pilot testing, we found no evidence of outside the cold chain implementation at national scale. This finding echoes the conclusions of Dadari et al., whose 2024 scoping review of use of outside the cold chain and controlled temperature chain in LMICs similarly noted that outside the cold chain approaches consistently improved operational flexibility and access but remained limited to small-scale pilots due to policy hesitancy, regulatory gaps, and uncertainty around integrating outside the cold chain vaccines into national guidelines [12]. Together, these findings suggest that outside the cold chain storage remains an underutilized but high-potential strategy that may require targeted implementation research and supportive policy mechanisms to move from pilot use to routine practice.

Within our review, evidence of CHWs serving as vaccinators spanned a range of documentation types. In addition to one empirical study describing CHW administration of birth dose vaccines in China [47], we identified a modeling analysis estimating potential coverage gains under scenarios where CHWs are authorized to vaccinate [44], as well as normative guidance and training materials from the WHO that explicitly identify CHWs as potential vaccinators for hepatitis B birth dose delivery [48,51]. This pattern is consistent with the broader global literature, where empirical implementation remains limited to a small number of country experiences, such as Burkina Faso and Malawi [58,59]. However, a recent global review by Gibson et al. found that among 75 documented CHWs programs, 20 permitted CHWs to administer vaccines, suggesting that while CHW-led vaccination is not widespread, it is an established practice in a meaningful subset of settings [11]. Collectively, this body of evidence indicates that, with appropriate training, supervision, and equipment, expanding CHW roles to include vaccination represents a feasible option that warrants further exploration.

While we considered cold chain approaches and health worker roles as separate secondary questions, our findings show that flexible cold chain approaches (e.g., OCC/CTC or CPAD packaging) and expanded CHW engagement as vaccinators may be mutually reinforcing. Several countries in Asia operationalized outside the cold chain/controlled temperature chain storage together with broader roles for community-based vaccinators, enabling rapid birth dose delivery during postnatal home visits and community follow-up [24,29,32,34,41]. Our review builds on prior evidence syntheses and responds to calls for research on home birth vaccination strategies [60]. Earlier reviews separately demonstrated the potential of (a) flexible cold chain strategies to extend reach [12] and (b) CHW task shifting to expand access in underserved populations [11]. In contrast, our rapid scoping review highlights how these strategies functioned together in practice, through CHWs and village-level providers conducting postnatal home visits and delivering hepatitis B birth dose using outside the cold chain storage or compact prefilled auto-disable devices.

These operational challenges driving low hepatitis B birth dose coverage are broadly similar across Asian and African settings according to studies in our review. Stockouts, inadequate cold chain capacity, difficult terrain and distance barriers to facility access, high rates of out-of-facility births, and missed vaccination opportunities for newborns who never reach a facility are documented across both regions. This shared operational reality suggests that the strategies documented in Asian settings, particularly outside the cold chain storage with CHW-led birth notification, referral, and vaccination, are conceptually relevant to delivery contexts in African settings facing the same underlying barriers. However, a critical difference lies not in operational challenges but in the policy and programmatic foundation within which these strategies are being implemented. As of 2025, only 19 of 47 African countries (40%) have introduced the hepatitis B birth dose into their national immunization programs, compared to 7 of the 10 (70%) in South-East Asia and all 28 Western Pacific countries [61]. Universal hepatitis B birth dose policy adoption within 24 h of birth follows a similar pattern, with 36% of African countries having adopted such policy compared to 83.3% in South-East Asia and 92.5% in the Western Pacific Region [62]. These policy gaps are reflected in coverage: WHO data show that hepatitis B birth dose coverage within 24 h of birth stands at 17% in Africa, compared to 56% in South-East Asia and 78% in the Western Pacific Region [63]. The Asian studies included in this review, particularly those from China, Lao PDR, and Kiribati, were conducted in settings where the hepatitis B birth dose was already embedded in the national immunization program, free vaccine policies were established, and community health worker roles in immunization delivery were already defined. Many African countries documented in this review are still working to establish these conditions, with the included studies highlighting persistent gaps in standard operating procedures, staff role clarity, 24/7 vaccine access, and policy frameworks for out-of-facility vaccination [28,41,45]. The strategies documented in this review therefore remain highly relevant to African settings, but their successful adoption will likely depend on concurrent progress in national policy development and health system strengthening alongside implementation of specific delivery strategies.

Several important areas for future research emerge from this review that would strengthen the evidence base for out-of-facility birth dose delivery. Further work is needed to understand how specific delivery models operate under real-world conditions, particularly where multiple components are deployed together. We hypothesize that delivery models that include all three components (identification, vaccine delivery, and uptake support) will be most successful. Greater attention to the interaction between system enablers/barriers and chosen strategies would help clarify why certain approaches succeed in some settings but falter in others, and how these configurations influence the timeliness of hepatitis B birth dose uptake. Context-specific assessments that identify which combinations of contextual factors and implementation strategies yield the highest timely coverage would be valuable for countries preparing to introduce the birth dose. In addition, there is a need for operational research that translates these insights into practical tools for implementers that can support countries as they adapt global experiences to their own system realities. Specifically, comparative effectiveness research examining outcomes across different combinations of identification, delivery, and uptake support strategies would clarify which configurations are most effective across different system contexts: a gap that limits countries’ ability to make evidence-based choices during introduction planning. Cost-effectiveness analyses, particularly for rural/remote and nomadic populations, would help programs assess the value of resource-intensive strategies such as home-based outreach relative to lower-cost referral approaches. Localized pilot studies evaluating feasibility and scale-up pathways for national rollout of outside the cold chain approaches can address the persistent gap between successful small-scale pilots and national adoption. Implementation fidelity assessments can illuminate how strategies are adapted in practice and what adaptations preserve or compromise effectiveness. Caregiver acceptability studies, particularly in high home-birth settings where cultural norms around newborn care are strong, can inform the design and demand-generation components to ensure people-centeredness is prioritized. Finally, sustainability evaluations of community-based models, including CHW retention, motivation, and performance in vaccine administration roles, can help determine whether these approaches can be maintained beyond initial pilot phases. Structured cross-country learning mechanisms will be equally important so that emerging lessons are rapidly shared, particularly among countries in early phases of introduction, helping reduce duplication of effort and accelerate adoption of what works.

Our review has several strengths, including its focus on delivery strategies themselves, presented to highlight their features and the system conditions shaping their feasibility. The inclusion of studies from diverse geographic and programmatic contexts broadens the applicability of the synthesis. However, this study also has limitations. The peer-reviewed literature search was limited to PubMed, which may have excluded relevant studies indexed in other databases, as described in Section 2. In addition, the search was limited to English-language sources and a targeted set of organizational websites, which may have excluded relevant evidence, especially programmatic materials or unpublished evaluations from non-English-speaking countries. Although screening and data extraction began with a calibration phase to ensure consistency, most of the process was conducted by a single reviewer, which introduces a risk of selection bias. As a scoping review, we did not assess study quality or evaluate the comparative effectiveness of strategies. Therefore, the results should be interpreted as a summary of existing evidence rather than an evaluation of its robustness. Finally, by centering specifically on delivery strategies for reaching babies born in out-of-facility settings, we may have missed insights from adjacent MNCH or immunization interventions that could be adapted for birth dose delivery. These limitations underscore the need for continued primary research, comparative evaluations, and implementation research studies to build on the strategic landscape mapped here.

The timing of this review is particularly relevant given that in June 2024, Gavi expanded eligibility to apply for Gavi support to introduce the hepatitis B birth dose vaccine to 38 countries [64]. At the same time, the broader global health financing landscape is shifting: many immunization and MNCH programs are navigating constrained budgets, competing priorities, and reductions or reallocation of external funding [65,66]. Donor transitions, funding cuts, and the increasing need for countries to co-finance or fully finance routine immunization services place additional pressure on governments to select delivery approaches that are cost-efficient, integrate well with existing platforms, and maximize the use of limited human and financial resources. By documenting how different out-of-facility implementation strategies have been designed and implemented, this review supports evidence-informed planning for hepatitis B birth dose introduction and scale-up where births outside health facilities are common, system constraints are significant, and timely coverage remains low.

## 5. Conclusions

This rapid scoping review shows that reaching newborns born outside health facilities with timely hepatitis B birth dose vaccination is operationally feasible but requires coordinated action across identification, caregiver engagement, and delivery components, supported by enabling health system conditions. This review describes a diverse range of strategies that have been implemented across LMICs, including flexible cold chain approaches and expanded roles for community-based providers, which remain underutilized at scale. As more countries, particularly in Africa, are moving towards hepatitis B birth dose introduction and scale-up, this review provides a summary of the evidence base to inform contextually appropriate delivery strategies.

## Figures and Tables

**Table 1:** Eligibility criteria details.

Categories	Inclusion	Exclusion
Population	Newborns delivered outside health facilities	Populations not involving newborns or out-of-facility births
Concept	Studies examining delivery approaches, service models, or implementation strategies for administering birth dose vaccines within 24 h of birth	Studies not focused on implementation or delivery strategies
Context	Out-of-facility births in LMICs, including rural or hard-to-reach areas	Studies solely describing facility-based delivery contexts
Geographic scope	LMIC setting	Non-LMIC setting
Time frame	Publication in the last 20 years (1 January 2005 to 1 April 2025)	Publications before 2005
Publication status	Peer-reviewed and non-peer-reviewed materials, including gray literature, program reports, policy documents, and collaborator-shared materials not publicly available	Materials lacking sufficient methodological or implementation detail
Study type/design	Implementation science, program evaluations/reports, case studies	Perspective or commentary articles without implementation focus
Vaccination strategies	A strategy or intervention to deliver any birth dose vaccine (BCG, OPV0, or hepatitis B) to newborns outside of health facilities as part of routine immunization efforts	No inclusion of birth dose vaccines; campaign-specific strategies (e.g., national or catch-up campaigns); determinants of vaccination
Language	English	Languages other than English

Abbreviations: BCG, Bacillus Calmette–Guérin; LMIC, low-/middle-income country; OPV0, oral polio vaccine birth dose.

**Table 2:** Detailed characteristics of the included studies.

Study No.	Citation	Source Type	Country/Region	Vaccine (s)	Health Worker Cadre (s)	Cold Chain/Packaging Approach	Out-of-Facility Strategy Type (s)	Summary of Delivery Strategy	Summary of Reported Outcomes
1	Ajayi et al., 2023 [28]	Conference presentation	Cameroon, Nigeria	Hepatitis B birth dose, OPV0	Not specified	Not specified	Integration with MNCH; community education	Interventions in subnational areas of Cameroon and Nigeria strengthened coordination between MNCH and RI units; addressed cold chain and reporting barriers; built capacity and knowledge of HCWs on birth dose delivery; and increased caregiver awareness.	In Cameroon, timely BCG and OPV0 vaccination among out-of-facility births increased substantially (from 5% to 26%, and 3% to 25% respectively). In Nigeria, timely birth dose vaccination within 24 h more than doubled during the intervention.
2	Allison et al., 2017 [29]	Peer-reviewed research article	Global	Hepatitis B birth dose	Midwife/TBA	OCC	Integration with MNCH; birth notifications and community tracking; flexible cold chain approach	An evaluation was conducted on the correlation between institutional delivery or skilled TBA rates and hepatitis B birth dose coverage, including in contexts using hepatitis B birth dose OCC.	Hepatitis B birth dose coverage was strongly correlated with skilled birth attendance (rho = 0.44, *p* < 0.001) and institutional delivery rates (rho = 0.42, *p* < 0.001). Increasing skilled deliveries, improving facility delivery rates, and using hepatitis B birth dose OCC where needed would raise coverage.
3	Breakwell et al., 2017 [30]	Peer-reviewed research article	Solomon Islands	Hepatitis B birth dose	Not specified	OCC	Flexible cold chain approach	A pilot study conducted storing hepatitis B birth dose vaccine OCC in Solomon Islands health facilities and home-birth settings.	Timely hepatitis B birth dose coverage increased from 30% to 68% among facility births and from 4% to 24% among home births. Temperature excursions above 37 °C were rare, but vaccine wastage was high and shortages common.
4	Breakwell et al., 2017 [31]	Peer-reviewed research article	Africa region	Hepatitis B birth dose	Not specified	Not specified	Outreach; task-shifting vaccinators; birth notification and community tracking; flexible cold chain approach	Regional review summarizing strategies implemented across African contexts, including policy development, maximizing hepatitis B birth dose coverage in facility births, training CHWs to vaccinate, and storing the vaccine OCC.	No outcomes of birth dose delivery strategies reported, although the review summarizes challenges and strategies for improving hepatitis B vaccine birth dose coverage in Africa.
5	Chang et al., 2019 [32]	Peer-reviewed research article	Global	Hepatitis B birth dose	Not specified	Not specified	Community education	Survey of HBV experts (63 countries) identifying obstacles and proposed strategies for reaching home births through supplying vaccines, training health workers, and educating policymakers.	No outcomes of birth dose delivery strategies reported. In 36% of countries—including all in Africa—the first dose was given after 24 h. The birth dose was unavailable for home births in 45% of countries (92% in Africa; 50% in Latin America).
6	Ethiopia Ministry of Health, 2022 [33]	Program report	Ethiopia	Hepatitis B birth dose	Facility-based worker, CHW, Midwife/TBA	Active, Passive	Outreach; postnatal home visit; integration with MNCH; birth notification and community tracking; task-shifting vaccinators	National pilot of hepatitis B birth dose program identifying strategies such as redesigning newborn tracking for home deliveries, integrating vaccination into newborn care, training TBAs, and equipping CHWs for PNC home visit vaccination.	88% of facilities had systems to track home deliveries, and some provided hepatitis B birth dose during PNC visits for babies up to 14 days old. Only 35% of families were referred by CHWs, and persistent health-system issues highlighted need for stronger systems for timely home-birth vaccination.
7	Farrenkopf et al., 2023 [34]	Conference presentation	Madagascar	BCG	Not specified	Not specified	Outreach; integration with MNCH	Context-specific strategies co-created in Madagascar proposed to improve access via outreach and integration of MNCH and immunization services.	Solutions to strengthen birth dose platforms were co-created, but no outcomes were reported.
8	Hipgrave et al., 2006 [35]	Peer-reviewed research article	Global with specific data from China, Indonesia, Vietnam	Hepatitis B birth dose	Midwife/TBA, village doctor	OCC	Flexible cold chain approach; task-shifting vaccinators	Studies in three countries piloted storing hepatitis B birth dose vaccines OCC, with TBAs administering vaccines in some sites.	All three studies concluded that hepatitis B birth dose vaccine can be stored OCC in semi-tropical temperatures without losing its potency.
9	Hutin et al., 2013 [36]	Peer-reviewed research article	China	Hepatitis B birth dose	Facility-based worker, village doctor	Not specified	Postnatal home visit; integration with MNCH; birth notification and community tracking; incentives; community education; referral/linkage to health facilities	Five demonstration projects in China used incentives for facility delivery to increase awareness of hepatitis B birth dose benefits, and tracked and notified home births to link them to vaccination.	Timely coverage of hepatitis B birth dose increased in all five demonstration projects.
10	Izudi et al., 2025 [37]	Peer-reviewed research article	Kenya	BCG, OPV0	Facility-based worker, CHW	Not specified	Mobile reminders; birth notification and community tracking; referral/linkage to health facilities	Text messages were sent to caregivers before and on the day of delivery to urge them to consider taking their babies for timely vaccination at health facilities.	Timely vaccination improved during the intervention, with increases of 16% for OPV, 14% for BCG, and 19% for receiving both vaccines within two weeks.
11	Kolwaite et al., 2016 [38]	Peer-reviewed research article	Lao People’s Democratic Republic	Hepatitis B birth dose	Not specified	OCC	Flexible cold chain approach	Pilot in Lao PDR storing hepatitis B birth dose vaccines OCC across village types and offering vaccination through fixed, outreach, and mobile services.	Hepatitis B birth dose coverage increased from 0% to 27% overall in the intervention arm, with the largest gains in fixed villages (33% to 57%), followed by outreach (0% to 27%) and mobile villages (0% to 6%). Among home births in fixed villages, coverage rose from 1% to 30%.
12	Levine et al., 2021 [39]	Peer-reviewed research article	Ghana	BCG, OPV0	CHW	Not specified	Outreach; birth notification and community tracking; mobile reminders; incentives	In rural Ghana, CHWs placed reminder calls after new births and provided incentives to caregivers and volunteers to promote timely vaccination.	Mobile phone reminders and small incentives improved timely early vaccination in rural communities, especially when CHWs delivered the intervention.
13	Li et al., 2017 [40]	Peer-reviewed research article	Republic of Kiribati	Hepatitis B birth dose	Facility-based worker, CHW, midwife/TBA	OCC	Birth notification and community tracking; flexible cold chain approach; outreach	In South Tarawa and outer islands, pregnant women received education, CHWs and health workers improved inter-communications, and vaccines were taken OCC to reach home births.	Hepatitis B birth dose coverage among home births increased from 70% to 84% (*p* = 0.1) in South Tarawa and 49% to 75% (*p* < 0.01) in the Outer Islands. Caregiver knowledge about hepatitis B and birth dose timing improved in the Outer Islands but not in South Tarawa.
14	Moturi et al., 2018 [41]	Peer-reviewed research article	Botswana, the Gambia, Namibia, Nigeria, and São Tomé and Príncipe	Hepatitis B birth dose	Facility-based worker	Not specified	Integration with MNCH; outreach	Knowledge, attitudes, and practices assessments in facilities in Africa Region proposed strategies such as developing standard operating procedures for timely health facility administration, and coordination between EPI and MNCH programs.	No outcomes of birth dose delivery strategies reported. Barriers to timely hepatitis B birth dose included unclear staff roles, poor integration with newborn care, delays at discharge, limited service availability, gaps in staff knowledge, absence of outreach for home births, and reporting tools that did not capture timeliness.
15	Okoli et al., 2014 [42]	Peer-reviewed research article	Nigeria	OPV0	Facility-based worker	Not specified	Incentives	A conditional cash transfer program targeting pregnant women in rural and underserved areas was piloted in primary health care facilities in Nigeria to incentivize use of ANC/PNC and skilled delivery.	The monthly number of women attending four or more ANC visits increased significantly (+15.12 per 100,000). Changes in skilled birth attendance and neonatal OPV at birth were not significant.
16	Sahito et al., 2020 [43]	Peer-reviewed research article	Pakistan	BCG	Facility-based worker	Not specified	Outreach; incentives; referral/linkage to health facilities; birth notification and community tracking	In Pakistan, trained and incentivized TBAs to enroll and refer children for vaccination at the health facility.	BCG coverage increased in both arms, with the highest increase (74%) in the TBA arm. TBAs continue to provide guidance on newborn vaccination, but they are less likely to go beyond routine efforts to ensure timely vaccination without monetary incentives, as they have no other source of income.
17	Seaman et al., 2020 [44]	Peer-reviewed research article	LMICs	Hepatitis B birth dose	Facility-based worker, CHW	CTC, CPAD, Active, Passive	Flexible cold chain approach; task-shifting vaccinators	The study modeled cost and coverage targets for hepatitis B birth dose vaccination by using the existing methodologies of facility-based cold chain administration, CTC storage for facility births, CTC for community births, and lastly, training of lay health workers with CPADs to administer vaccines in the community.	Modeled coverage increased from 44% to 65% under current protocols, with maximum coverage ranging from 49% in sub-Saharan Africa to 79% in East/Southeast Asia and Oceania. Achieving 90% coverage required combining CTC and CPAD approaches. Optimized strategies were cost-saving and could avert 36.3 million DALYs, though require $494 million annually.
18	Solomon-Rakiep et al., 2024 [45]	Peer-reviewed research article	Africa Region	Hepatitis B birth dose	Not specified	Not specified	Outreach; postnatal home visit	Qualitative systematic review across 12 African countries summarizing practices for reaching home births through fixed sites, advanced outreach, and mobile outreach.	No birth dose outcomes were reported. The review highlighted cross-cutting factors affecting timely hepatitis B birth dose delivery, including policy clarity, supply and cold chain capacity, financing, HCW capability, monitoring gaps, contextual barriers, and maternal knowledge.
19	Uganda Ministry of Health, 2024 [46]	Gavi, the Vaccine Alliance application	Uganda	Hepatitis B birth dose	Facility-based worker, midwife/TBA	Active, Passive, Hybrid	Outreach; integration with MNCH; task-shifting vaccinators; birth notification and community tracking; referral/linkage to health facilities	Application describing proposed national strategies including task-shifting vaccinations to midwives, engaging CHWs for home birth tracking, and outreach vaccination.	No outcomes of birth dose delivery strategies reported, as this is an application.
20	Wang et al., 2007 [47]	Peer-reviewed research article	China	Hepatitis B birth dose	CHW	OCC, CPAD	Outreach; flexible cold chain approach; task-shifting vaccinators	The vaccine was stored OCC and administered by village-based health workers to infants in their homes using auto-disable syringes or CPADs.	Among home births, hepatitis B birth dose coverage increased sharply with OCC storage (+56.5%) and even more with CPADs under OCC (+70.5%). Coverage was higher when infants were vaccinated at home (73.5%) versus requiring parents to bring infants to township hospitals for vaccination (32.8%).
21	Wiesen et al., 2016 [48]	Peer-reviewed research article	Papua New Guinea	Hepatitis B birth dose	Facility-based worker, CHW, midwife/TBA	Not specified	Outreach; community education	Strategies were discussed based on existing barriers to hepatitis B birth dose reaching home deliveries and included outreach vaccination and educating pregnant women on the importance of timely BD and delivery in-facility.	Timely hepatitis B birth dose coverage was much higher in facilities (62%) than for home births (21%). Only 16% of caregivers had heard of the hepatitis B birth dose vaccine.
22	World Health Organization, 2015 [49]	Program report	Global	Hepatitis B birth dose	Facility-based worker, CHW, midwife/TBA	Active, Passive, Hybrid, CTC	Outreach; postnatal home visit; integration with MNCH; birth notification and community tracking; task-shifting vaccinators	The document outlines multiple strategies for increasing timely hepatitis B birth dose delivery among home births, including strategies for bringing newborns to a health facility and reaching newborns at home.	No outcomes of birth dose delivery strategies reported, as this is a guidance document.
23	World Health Organization, 2018 [50]	Policy document	Global	Hepatitis B birth dose	Not specified	Not specified	Outreach; referral/linkage to health facilities; flexible cold chain approach	The WHO recommends strategies for improving hepatitis B birth dose coverage, including promoting facility deliveries, outreach to home births, and OCC storage.	No outcomes of birth dose delivery strategies reported.
24	World Health Organization, 2024 [51]	Training package	Global	Hepatitis B birth dose	CHW, midwife/TBA	Passive	Postnatal home visit; task-shifting vaccinators	Updated WHO guidance recommending that skilled TBAs and PNC providers administer hepatitis B birth dose during PNC home visits.	No outcomes of birth dose delivery strategies reported.
25	Wu et al., 2015 [52]	Peer-reviewed research article	China	Hepatitis B birth dose	Midwife/TBA	Not specified	Integration with MNCH	National policy rollout in China from 2003 provides the hepatitis B birth dose vaccine free of cost.	Timely hepatitis B birth dose initiation rose to 91.4% among children born in 2005, up from 40–60% in earlier cohorts. Children born in 2003–2005 were 2.6 to 9 times more likely to receive the birth dose on time than those born in 1996–2001.
26	Xeuatvongsa et al., 2016 [53]	Peer-reviewed research article	Lao People’s Democratic Republic	Hepatitis B birth dose	Facility-based worker	Active	Outreach; postnatal home visit; incentives; mobile reminders	Intervention in Lao PDR providing CHWs mobile phones and per diems to support real-time birth notification and PNC home visits.	Hepatitis B birth dose coverage (within 30 days) increased in both intervention and comparison areas, with higher coverage among facility births than home births. Among home births, coverage improved regardless of whether a PNC home visit occurred.

Abbreviations: ANC, antenatal care; BCG, bacillus Calmette–Guérin; CHW, community health worker; CPAD, compact prefilled autodisable device; CTC, controlled temperature chain; EPI, Expanded Program on Immunization; HBV, hepatitis B virus; HCW, health care worker; MNCH, maternal, newborn, and child health; OCC, outside the cold chain; OPV0, oral polio vaccine birth dose; PDR, People’s Democratic Republic; PNC, postnatal care; RI, routine immunization; TBA, traditional birth attendant. Definitions: active cold chain, using powered systems like refrigeration for continuous temperature control; passive cold chain, reliance on pre-conditioned coolants like ice packs in insulated containers for temperature control without external power sources [54].

**Table 3:** Birth dose vaccine delivery strategies to reach newborns delivered outside of health facilities.

Delivery Strategy Components	Sub-Components	Examples from Selected Studies (Geography)
Identify and track home births	Register pregnant women	Community volunteers keep pregnancy registers that they share with health facilities (Papua New Guinea, Kiribati) [40,48] Two lay CHWs from each village register all pregnant women in their 2nd or 3rd trimester in a central database using a mobile phone (Kenya) [37]
	Notify facilities of home births	Community volunteers inform facility health workers of home births (Papua New Guinea, Ethiopia, Lao People’s Democratic Republic [PDR], Pakistan) [31,33,43,48] Village health volunteers receive mobile phones and phone credits to call health care workers for imminent delivery or birth notification (Lao PDR) [53]
	Perform active case finding and referral	Village health teams actively seek out and refer newborns for vaccination (Uganda) [46]
Administer hepatitis B birth dose vaccine to newborns within 24 h of birth	Enable health workers to vaccinate children in their homes	Health facilities conduct outreach for home births (Papua New Guinea) [48] Health workers travel to vaccinate children born at home (Ethiopia) [33]
	Integrate or coordinate with maternal postnatal care for home-based administration	Postnatal care providers (midwives, nurses, or skilled birth attendants) administer birth dose vaccines during postnatal care home visits (Uganda, Global) [46,49,51]
	Train community cadres to administer hepatitis B birth dose at home	Community cadres, such as CHWs, midwives, or village doctors or nurses, are trained to administer hepatitis B birth dose to newborns at home (Uganda, Global, Pakistan) [41,43,46,49,51]
	Provide transport to bring the newborn to the health facility for vaccination	Funding or supplement transportation is provided to bring mothers and babies to a facility for birth dose vaccination (Global) [49]
Provide education or incentives to encourage birth dose vaccination	Educate caregivers about the importance and timing of hepatitis B birth dose	Village health volunteers or village doctors recommend timely birth dose vaccination to pregnant women (Papua New Guinea, Kiribati, China) [40,47,48] ANC care visits are used to educate pregnant women on Hepatitis b birth dose (Global, Botswana, the Gambia, Sao Tome and Principe) [49,51] Traditional birth attendants raise awareness on hepatitis B birth dose and refer mothers for birth dose vaccination (Pakistan) [43] Information, education, and communication interventions are used to increase population awareness for birth dose vaccination (China) [47]
	Send mobile nudges or reminders	Mobile nudges (voice call reminders) are used, providing general information on the importance of birth dose vaccines and personalized information on where and when vaccine services are available (through outreach or fixed sites) (Ghana) [39] Automated text message reminders are sent to mothers (a) upon registration during pregnancy, (b) two weeks before the expected delivery date, and (c) on the expected delivery date, with each message reminding them to consider taking their newborn for timely vaccination (Kenya) [37]
	Offer incentives to caregivers	Pregnant women are enrolled in a conditional cash transfer program that pays them to attend ANC, skilled delivery, and postnatal care, including birth dose vaccination (Nigeria) [42] Mothers are given a mobile money reward if their child was vaccinated on time with BCG and OPV0 (Ghana) [39]
	Offer incentives to community health care providers	Traditional birth attendants are given an incentive for each complete child vaccination they have referred (Pakistan) [43] Community health volunteers are given a mobile money reward for each verified, on-time vaccination of BCG and OPV0 in their communities (Ghana) [39]

Abbreviations: ANC, antenatal care; CHW, community health worker; OPV0, oral polio vaccine birth dose.

**Table 4:** Enablers and barriers influencing out-of-facility birth dose vaccine delivery strategies.

Factors (Enabler, Barrier, or Mixed Evidence)	Reference Evidence (Geography)
National level	
Political commitment (enabler)	Educating policymakers to implement and/or improve vaccine programs (e.g., via campaigns from international medical societies) may help overcome barriers to increasing hepatitis B coverage (Global) [32] Strong political commitment is essential to allocate funding for hepatitis B birth dose, and advocacy with partners in related sectors (e.g., cancer prevention, chronic disease prevention, newborn care) may strengthen political commitment (Africa Region) [31] HBV is a priority health issue in the country and there is strong support to implement hepatitis B birth dose (Botswana, the Gambia, Namibia, Nigeria, Sao Tome and Principe) [41]
Partner collaboration (enabler)	Advocacy by physician/nurses associations, the private sector, hepatology associations, and other stakeholders enabled hepatitis B birth dose introduction (Nigeria, Sao Tome and Principe) [41] Collaboration with international, national, and local partners provided opportunities to resolve implementation issues (China) [36]
COVID-19 disruptions (barrier)	The COVID-19 pandemic caused disruptions and reprioritization, taking precedence over hepatitis B birth dose, which led to delays in implementing the birth dose pilot (Cameroon) [28]
Clear national policies (mixed)	Unclear policy on providing vaccination at home births is a constraint to timely BCG vaccination (Madagascar) [34] A national policy that assigns clear vaccine administration responsibility (“the one who delivers the child administers the vaccine”) is an enabler of timely hepatitis B birth dose coverage (China) [36] Integrated EPI and MNCH policies facilitated collaboration on hepatitis B birth dose implementation (Sao Tome and Principe) [41]
Infrastructure and connectivity (barrier)	Poor local mobile network coverage and challenges reaching women who did not own personal phones were barriers to using voice call reminders to encourage mothers to bring their children for vaccination (Ghana) [39]
Health system level	
Immunization service delivery schedule (mixed)	The limited availability of vaccination services (often half a day on weekdays and not on weekends or public holidays) is a barrier to increasing hepatitis B birth dose coverage (Botswana, the Gambia, Namibia, Nigeria, Sao Tome and Principe) [41] Defining a detailed and tailored schedule to ensure 24/7 access to context-appropriate vaccine storage options, including after hours and during weekends, contributed to increased birth dose coverage (Cameroon, Nigeria) [28]
EPI and MNCH coordination or integration of services (enabler)	A multi-stakeholder task team, with participation from EPI and MNCH, enabled sustainability and joint accountability at national and subnational levels (Cameroon, Nigeria) [28] Integrating routine immunization services as part of the maternal and newborn package of care (e.g., RI stand in maternity unit, referrals) was key to improving timely birth dose administration (Nigeria) [28] EPI and MNCH staff joint micro-planning for birth dose administration for home births contributed to improvements in hepatitis B birth dose coverage (China) [36]
Linkages between facility and community levels of the health system (enabler)	Regular meetings are held between village health volunteers and facility HCWs to discuss pregnancy registration and outreach visits for home births (Papua New Guinea, Kiribati) [40,48] Village health volunteers share a list of pregnant women with facilities monthly (Kiribati) [40] Supervision of skilled birth attendants’ work includes checks of vaccination practices (Global) [49] Mobile phones and phone credit are provided to support village health volunteers in notifying HCWs of home births (Lao People’s Democratic Republic) [53] Supportive supervision is targeted to low birth dose coverage areas to address challenges (China) [36] A micro-plan is created for each preregistered pregnant woman, including estimated time/place of delivery, how the vaccine would be obtained, and who would administer it (China) [36]
Provider knowledge on birth dose administration (mixed)	Insufficient knowledge, training, and supervision among HCWs and village health volunteers regarding HBV and hepatitis B birth dose is a barrier to increasing hepatitis B birth dose coverage (Papua New Guinea) [48] HCWs report a lack of training for hepatitis B birth dose, but despite this there is high knowledge regarding HBV (Botswana, the Gambia, Namibia, Nigeria, Sao Tome and Principe) [41] In Nigeria, HCW lack of awareness about appropriate age limits for vaccine eligibility resulted in missed opportunities for vaccination (Nigeria) [41] HCW concerns about adverse events, multiple vaccine administrations, and administering birth doses to low-birth-weight children can impede timely coverage of birth dose vaccines (Cameroon, Nigeria) [28] Capacity-building and improvement in HCW knowledge on birth dose administration (including safety information and directly addressing hesitancy related to opening a multi-dose vaccine vial) contributed to increased birth dose coverage (Cameroon) [28] HCW reluctance to vaccinate underweight or preterm newborns resulted in missed opportunities for timely hepatitis B birth dose vaccination (Global) [50] Outlining detailed roles and responsibilities for each HCW involved in birth dose vaccinations contributed to increased birth dose coverage (Cameroon, Nigeria) [28] Developing facility-specific workflows to train and orient HCWs on how birth dose administration fits into maternal and newborn care contributed to increased birth dose coverage (Cameroon, Nigeria) [28]
Policy implementation (mixed)	Availability of hepatitis B birth dose policies on-site led to higher hepatitis B birth dose coverage (Philippines) [31] The policy of maternal screening and selective hepatitis B birth dose vaccination was a barrier to achieving high hepatitis B birth dose coverage (Sao Tome and Principe) [41] A lack of standard operating procedures to explain when and where hepatitis B birth dose should be administered after birth was a barrier to increasing hepatitis B birth dose coverage (Botswana, the Gambia, Namibia, Nigeria, Sao Tome and Principe) [41]
Vaccine packaging, supply, and cold chain (mixed)	Stockouts cause constraints to birth dose vaccine delivery (Madagascar, Solomon Islands) [30,34] The lack of a cold chain to store the hepatitis B birth dose can limit vaccine delivery, especially for home births (Papua New Guinea, Global) [29,48] Ensuring 24/7 access to vaccines, including after hours and during the weekends, contributes to increased birth dose coverage (Cameroon, Nigeria) [28] Single-dose vials or compact prefilled auto-disable devices may be preferable for home births since compact prefilled auto-disable devices are easier to use and require less training (Global) [49] Market authorization for outside the cold chain was not a government priority given the resources required, which could go to interventions providing direct public health benefits (China) [36] Countries may be reluctant to allow outside the cold chain storage of hepatitis B vaccine because manufacturers do not currently provide stability data on their products and there are no published data linking vaccine vial monitor status and hepatitis B vaccine potency (Global) [35] Outside the cold chain is not yet widely used, largely due to the lack of a hepatitis B vaccine that has been licensed for use outside the cold chain (Global) [50]
Health system resources, costs (barrier)	The lack of transportation, staff, and funding is a barrier to outreach vaccination for home births (Papua New Guinea) [48] Community agents who can identify home births are not compensated, which is a key barrier to increasing birth dose coverage (Madagascar) [34]
Data, monitoring, and reporting (mixed)	Unclear denominators for areas with nomadic populations can make it difficult to estimate vaccine coverage (Botswana, the Gambia, Namibia, Nigeria, Sao Tome and Principe) [41] Poor data recording and reporting is a barrier to high hepatitis B birth dose coverage (Papua New Guinea) [48] Maternity registers do not have a dedicated column to record hepatitis B date of administration (the Gambia, Namibia, Nigeria, Sao Tome and Principe) [41] EPI reporting tools do not distinguish between hepatitis B birth dose delivered within 24 h vs. after 24 h, and district and national coverage calculations did not specify the timeliness of vaccination (Botswana, Namibia, Nigeria, Sao Tome and Principe) [41] CHWs and health facilities receive bimonthly reports about newborns due for vaccination (based on pregnancy registers) to plan for vaccination services (Kenya) [37] Few facilities analyze immunization data and use findings for programmatic planning (Botswana, the Gambia, Namibia, Nigeria, Sao Tome and Principe) [41] Development of a newborn data tool to complement existing health facility data tools helped to ensure birth dose vaccines are properly recorded (Cameroon) [28] A lack of data on specific barriers and facilitators to intervention uptake was a missed opportunity to inform scalability (Kenya) [37]
Community level	
Mother/caregiver awareness of birth dose vaccines (mixed)	Low awareness among caregivers of recommendations for birth dose vaccination is a key constraint (Madagascar) [34] There is low caregiver knowledge of HBV, complications, transmission routes, and hepatitis B vaccine (Papua New Guinea) [48] Hepatitis B birth dose vaccination is a highly acceptable intervention and there are no observed caregiver refusals (Botswana, the Gambia, Namibia, Nigeria, Sao Tome and Principe) [41] Caregiver concerns about adverse events and multiple administrations need to be addressed in a culturally appropriate manner to increase hepatitis B birth dose coverage (Cameroon, Nigeria) [28]
Cultural norms and beliefs (barrier)	According to some local customs, newborns cannot be taken out of the home during their first month (China, Africa Region) [45,47] Among the Hui of Ningxia, China, it is believed to be culturally inappropriate for strangers to see a newborn, which was a barrier to timely birth dose vaccination in homes [36] Cultural or religious practices (e.g., naming ceremonies, male circumcision) delay uptake of the hepatitis B birth dose (Gambia, Nigeria, Africa Region) [41,45]
Community engagement (mixed)	Health promotion activities (e.g., education during ANC care visits, handouts, radio campaigns) improved acceptance of vaccination among local communities (Indonesia) [45] Information, education, and communication efforts increased knowledge, which may have contributed to high birth dose coverage. However, they were limited by a top-down approach, complex language, and lack of interpersonal interactive communication activities and may miss marginalized populations (China) [36] Rumors and media reports of AEFIs result in hesitancy in caregivers and HCWs to vaccinate newborns with a hepatitis B birth dose, requiring a timely health risk communication response to maintain public confidence in the vaccine (Global) [50]
Costs to families (barrier)	The cost of transportation to the vaccination site and competing priorities (e.g., work) are key constraints to birth dose coverage (Madagascar) [34] Vaccine cost is a barrier, especially among low socioeconomic households—for example, the increase in hepatitis B birth dose coverage rates after implementation of the free-vaccine policy in China in 2003 (China) [52]

Abbreviations: AEFI, adverse event following immunization; ANC, antenatal care; CHW, community health worker; EPI, Expanded Program on Immunization; HBV, hepatitis B virus; HCW, health care worker; MNCH, maternal, newborn, and child health.

## Data Availability

The data charted as part of this rapid scoping review are derived from publicly available sources. Extracted data supporting the findings of this study are included in the article and Supplementary Materials. The data charting form is available from the corresponding author upon reasonable request.

## Supplementary Materials

The following supporting information can be downloaded at https://www.mdpi.com/article/10.3390/vaccines14070554/s1: Table S1: Preferred Reporting Items for Systematic reviews and Meta-Analyses extension for Scoping Reviews (PRISMA-ScR) Checklist; S2: Rapid scoping review protocol; S3: PubMed search strategy; Table S4. Summary of targeted website searches and gray literature sources reviewed; Table S5. Data extraction form.
