# Supplementary material for: Getting the Hepatitis B Birth Dose Vaccine to Every Baby: A Rapid Scoping Review of Birth Dose Vaccine Delivery Strategies in Out-of-Facility Settings
Source: Vaccines (Basel). 2026 Jun 24;14(7):554. doi: 10.3390/vaccines14070554 (PMC13418725; doi:10.3390/vaccines14070554)
Supplement: Supplementary file 1 [file vaccines-14-00554-s001.zip › vaccines-4346879-supplementary.pdf]

Supplemental Materials

Table S1. Preferred Reporting Items for Systematic reviews and Meta-Analyses extension for Scoping Reviews (PRISMA-ScR) Checklist

SECTION	ITEM	PRISMA-ScR CHECKLIST ITEM	REPORTED ON PAGE #
**TITLE**			
Title	1	Identify the report as a scoping review.	1
**ABSTRACT**			
Structured summary	2	Provide a structured summary that includes (as applicable): background, objectives, eligibility criteria, sources of evidence, charting methods, results, and conclusions that relate to the review questions and objectives.	1
**INTRODUCTION**			
Rationale	3	Describe the rationale for the review in the context of what is already known. Explain why the review questions/objectives lend themselves to a scoping review approach.	2
Objectives	4	Provide an explicit statement of the questions and objectives being addressed with reference to their key elements (e.g., population or participants, concepts, and context) or other relevant key elements used to conceptualize the review questions and/or objectives.	2-3
**METHODS**			
Protocol and registration	5	Indicate whether a review protocol exists; state if and where it can be accessed (e.g., a Web address); and if available, provide registration information, including the registration number.	3
Eligibility criteria	6	Specify characteristics of the sources of evidence used as eligibility criteria (e.g., years considered, language, and publication status), and provide a rationale.	3-4
Information sources*	7	Describe all information sources in the search (e.g., databases with dates of coverage and contact with authors to identify additional sources), as well as the date the most recent search was executed.	4
Search	8	Present the full electronic search strategy for at least 1 database, including any limits used, such that it could be repeated.	Supplementary Material S3
Selection of sources of evidence†	9	State the process for selecting sources of evidence (i.e., screening and eligibility) included in the scoping review.	5
Data charting process‡	10	Describe the methods of charting data from the included sources of evidence (e.g., calibrated forms or forms that have been tested by the team before their use, and whether data charting was done independently or in duplicate) and any processes for obtaining and confirming data from investigators.	5

SECTION	ITEM	PRISMA-ScR CHECKLIST ITEM	REPORTED ON PAGE #
Data items	11	List and define all variables for which data were sought and any assumptions and simplifications made.	Table S5
Critical appraisal of individual sources of evidence§	12	If done, provide a rationale for conducting a critical appraisal of included sources of evidence; describe the methods used and how this information was used in any data synthesis (if appropriate).	Not applicable
Synthesis of results	13	Describe the methods of handling and summarizing the data that were charted.	5
**RESULTS**			
Selection of sources of evidence	14	Give numbers of sources of evidence screened, assessed for eligibility, and included in the review, with reasons for exclusions at each stage, ideally using a flow diagram.	5-6
Characteristics of sources of evidence	15	For each source of evidence, present characteristics for which data were charted and provide the citations.	6-13
Critical appraisal within sources of evidence	16	If done, present data on critical appraisal of included sources of evidence (see item 12).	Not applicable
Results of individual sources of evidence	17	For each included source of evidence, present the relevant data that were charted that relate to the review questions and objectives.	7-13
Synthesis of results	18	Summarize and/or present the charting results as they relate to the review questions and objectives.	13-19
**DISCUSSION**			
Summary of evidence	19	Summarize the main results (including an overview of concepts, themes, and types of evidence available), link to the review questions and objectives, and consider the relevance to key groups.	19-23
Limitations	20	Discuss the limitations of the scoping review process.	23
Conclusions	21	Provide a general interpretation of the results with respect to the review questions and objectives, as well as potential implications and/or next steps.	24
**FUNDING**			
Funding	22	Describe sources of funding for the included sources of evidence, as well as sources of funding for the scoping review. Describe the role of the funders of the scoping review.	24

JBI = Joanna Briggs Institute; PRISMA-ScR = Preferred Reporting Items for Systematic reviews and Meta-Analyses extension for Scoping Reviews.

* Where *sources of evidence* (see second footnote) are compiled from, such as bibliographic databases, social media platforms, and Web sites.

† A more inclusive/heterogeneous term used to account for the different types of evidence or data sources (e.g., quantitative and/or qualitative research, expert opinion, and policy documents) that may be eligible in a scoping review as opposed to only studies. This is not to be confused with *information sources* (see first footnote).

‡ The frameworks by Arksey and O'Malley (6) and Levac and colleagues (7) and the JBI guidance (4, 5) refer to the process of data extraction in a scoping review as data charting.

§ The process of systematically examining research evidence to assess its validity, results, and relevance before using it to inform a decision. This term is used for items 12 and 19 instead of "risk of bias" (which is more applicable to systematic reviews of interventions) to include and acknowledge the various sources of evidence that may be used in a scoping review (e.g., quantitative and/or qualitative research, expert opinion, and policy document).

From: Tricco AC, Lillie E, Zarin W, O'Brien KK, Colquhoun H, Levac D, et al. PRISMA Extension for Scoping Reviews (PRISMAScR): Checklist and Explanation. Ann Intern Med. 2018;169:467–473. doi: 10.7326/M18-0850.

S2. Rapid scoping review protocol

Approach summary

The rapid scoping review aims to identify and synthesize evidence on immunization delivery strategies that facilitate the administration of a birth dose (BD) vaccine within 24 hours of birth for out-of-facility births in low- and middle-income countries (LMIC). The results of the rapid scoping review will inform the design of out-of-facility vaccination delivery strategies for birth dose (BD) of hepatitis B (HepB), which will be developed through a human-centered design prototyping process and subsequently tested through implementation science approaches as part of the learning agenda.

Research questions

The primary research questions for the scoping review are:

- What strategies have been used to deliver BD vaccines to newborns delivered outside of health facilities in LMIC?
- What are the successes, challenges, and lessons learned from implementing out-of-facility strategies?

In addition, we have identified two secondary research questions for understanding aspects of the cold chain and human resources for health required to support vaccine delivery strategies:

- What cold chain approaches, including out of cold chain or controlled temperature chain, have been used to enable out-of-facility BD delivery strategies in LMIC?
- What type(s) of health workers, including non-physician and community cadres (inclusive of community health workers [CHWs]), have implemented the out-of-facility BD delivery strategies (including vaccine administration) in LMIC?

Methods

PATH selected a scoping review because we aim to systematically identify and map the breadth of evidence available on what strategies are being used to deliver BD vaccines to newborns delivered outside of health facilities in LMIC. Due to the compressed timeline to inform planned HepB-BD introduction across countries, we are undertaking a rapid scoping review. The review will work from a health systems perspective and focus on delivery strategies for routine immunization efforts. This review will be conducted in accordance with the Joanna Briggs Institute methodology for scoping reviews.

Eligibility criteria

Using the Population, Concept, and Context framework to describe the focus of the scoping review, the eligibility criteria are as follows (see Table SA1 for further criteria):

- **Population**: newborns delivered outside of health facilities in LMIC.
- **Concept**: immunization delivery strategies aimed at administering a BD vaccine within 24 hours of birth.
- **Context**: out-of-facility births in LMIC, particularly in rural or hard-to-reach areas.

Table SA1. Additional inclusion/exclusion criteria.

Categories	Inclusion	Exclusion
Geographic scope	LMIC setting	Non-LMIC setting
Time frame	Publication in the last 20 years (January 1, 2005, to April 1, 2025)	Pre-2005 publication
Study type / design	Implementation science, program evaluations/reports, case studies	Perspective articles or studies without a focus on implementation or evaluation
Vaccination strategies	A strategy or intervention to deliver birth-dose vaccines (BCG, OPV0, or HepB) to newborns outside of health facilities as part of routine immunization efforts	No inclusion of birth dose vaccines; campaign-specific strategies (e.g., national or catch-up campaigns); determinants of vaccination
Language	English	Language other than English

Abbreviations: BCG, Bacillus Calmette–Guérin; Hep B, hepatitis B; LMIC, low- and middle-income countries; OPV0, oral polio vaccine birth dose.

Search strategy

A comprehensive search for resources published between 2005 and the present will be conducted in the PubMed database. The PubMed search will be supplemented by a targeted search of relevant websites (Gavi, World Health Organization, Technet-21, and the Boost community) to capture recent practices and innovations in out-of-facility delivery strategies published during the last ten years, from 2015 to present.

Search terms

A combination of medical subject heading terms and free-text words will be used (Table SA2), adapted to the specific database requirements. Medical subject heading terms and keywords will be combined using Boolean operators (AND/OR). Based on preliminary searches we may refine or expand the search terms.

Table SA2. Search terms used.

	MeSH terms	Free text terms
**Population**	“Infant, Newborn”<br>“Perinatal Care”<br>"Home Childbirth”<br>“Midwifery”	“Out-of-facility birth*” [tw]<br>“Out-of-facility deliver*” [tw]<br>“Home birth*” [tw]<br>“Community birth*” [tw]<br>“Traditional birth*”
**Concept**	“Immunization”<br>“Vaccination”<br>“Immunization Programs”<br>“Maternal-Child Health Services”<br>“Vaccination Coverage”<br>“Immunization Schedule”<br>“Postnatal Care”<br>“Delivery of health care”	“Birth dose*” [tw]<br>“Birth-dose*” [tw]<br>“Birthdose*” [tw]<br>“Neonatal vaccin*” [tw]<br>“Mobile vaccin*” [tw]<br>“Outreach vaccin*” [tw]<br>“Community vaccin*”[tw]
**Context**	“Developing Countries”<br>“Global Health”<br>“Africa”<br>“Sub-Saharan Africa”<br>“Rural Health Services”	“Low- and middle-income countr*”<br>“LMIC*”<br>“Africa*”<br>“Resource limited”<br>“Resource-limited”

	MeSH terms	Free text terms
	"Rural population"	"Resource constrained"<br>"Remote"<br>"Hard to reach"
**Specific vaccines**	"Hepatitis B Vaccines"<br>"BCG Vaccine"<br>"Poliovirus Vaccine, Oral"	"Hep B"<br>"BCG"<br>"OPV"

Abbreviations: BCG, Bacillus Calmette–Guérin; LMIC, low- and middle-income countries; MeSH, medical subject heading; OPV, oral polio vaccine.

Study selection

The study selection steps are as follows:

1. *Title and abstract screening*: Two independent reviewers will screen 20 percent of studies for relevance based on eligibility criteria using Covidence. We then will assess reviewer agreement for this proportion of studies, and if agreement is high (≥ 0.8), we will proceed with single screening.**Error! Bookmark not defined.**
2. *Full-text review*: Selected studies will undergo a full-text review by one reviewer. This is necessary given the compressed timeline for this rapid review. To mitigate bias from a single reviewer, the two independent full-text reviewers will meet at least once a week to discuss progress and raise questions to support a standardized approach.
3. *Data extraction*: Key data will be extracted using a standardized template in Covidence. The PICO [Population, Intervention, Comparison, Outcome] Framework will be used to inform the data elements for extraction. An illustrative list of data elements for extraction is included in Table SA3.

Table SA3. Data elements for extraction

Domain	Data element	Description
General study information	Lead author	Study author
	Title	Study title
	Publication year	Study publication year
	Study setting: Country	(Can select multiple countries)
	Study setting: Urban/rural	Urban, peri-urban, rural, not applicable, or not specified
	Study design	Qualitative research study, quantitative research study, mixed-methods research study, case study, program report, policy document/guideline, other
Population	Target population	Newborns, mothers, caregivers, TBAs, CHWs, not specified, other (can select multiple populations)
	Birth setting	Institution, home birth, community birth, other out-of-facility locations, not specified (can select multiple settings)
Vaccine	Vaccine	BCG, OPV0, HBV, other (can select multiple vaccines)
	Vaccine presentation	Single-dose vial, multidose vial, other, not specified
	Delivery strategy description	(Open text)

Domain	Data element	Description
Intervention (delivery strategy)	Delivery strategy type	Mobile outreach, PNC home visit, integration with maternal/newborn care, incentives, digital tracking, etc.*
	Implementation lessons	Implementation considerations, including successes, challenges, and lessons learned (open text)
	Adaptations	Description of what is unique about this strategy and what adaptations were made to the status quo (open text)
	Health workers involved	Facility health worker, CHW, TBA, outreach team, lay vaccinator, other
	Health worker who was the vaccinator	Facility health worker, CHW, TBA, outreach team, lay vaccinator, other
	Health worker lessons	Any information about health worker considerations (challenges, successes, lessons) for the delivery strategy (open text)
	Vaccine handling	Information about vaccine handling, such as the following questions (open text): How are vaccines stored (e.g., vaccine carriers)? Is coolant used (e.g., ice packs, cold water packs)? What is the time frame for vaccines being OCC (if applicable)? Are temperature monitoring tools mentioned, including a vaccine vial monitor?
	Cold chain lessons	Information about cold chain considerations (challenges, successes, lessons) for the delivery strategy (open text)
Comparison	Alternative strategies / status quo	Facility-based immunization, no intervention, other
Outcomes	Outcomes of strategy	(Open text)
	Type of outcome	Coverage, timeliness, equity, health impact, other

* We may refine these options during the initial screening.
Abbreviations: BCG, Bacillus Calmette–Guérin; CHW, community health worker; HBV, hepatitis B virus; OCC, out of cold chain; OPV0, oral polio vaccine birth dose; PNC, postnatal care; TBA, traditional birth attendant.

Data analysis and reporting

Identified delivery strategies will be summarized in tables organized according to relevant characteristics, including type of strategy, geography, vaccine(s) delivered, health workers involved, and vaccine handling used. Successes, challenges, and lessons learned associated with each strategy will be summarized. Gaps in evidence will be highlighted.

The rapid scoping review will be developed into a report (slide deck format) for sharing key insights. The guidelines from PRISMA-ScR (Preferred Reporting Items for Systematic reviews and Meta-Analyses extension for Scoping Reviews) will be used to inform reporting of the rapid scoping review rationale, methods, and results.[Error! Bookmark not defined.] Reporting visuals may include a:

- PRISMA-ScR figure to summarize the number of articles screened and reviewed.
- Map of the number of articles by country.
- List or table of delivery strategies organized according to relevant characteristics, including type of strategy, geography, vaccine(s) delivered, health workers involved, and cold chain used.

S3. PubMed search strategy

Population

("infant, newborn"[MeSH Terms] OR "Perinatal Care"[MeSH Terms] OR "Home Childbirth"[MeSH Terms] OR "Midwifery"[MeSH Terms] OR ("out-of-facility"[Text Word] AND "birth*"[Text Word]) OR ("out-of-facility"[Text Word] AND "deliver*"[Text Word]) OR "home birth*"[Text Word] OR "community birth*"[Text Word] OR "traditional birth*"[Text Word])

AND

Concept

("vaccination"[MeSH Terms] OR "immunization"[MeSH Terms] OR "vaccination"[MeSH Terms] OR "immunization programs"[MeSH Terms] OR "vaccination coverage"[MeSH Terms] OR "immunization schedule"[MeSH Terms] OR "maternal child health services"[MeSH Terms] OR "postnatal care"[MeSH Terms] OR "delivery of health care"[MeSH Terms] OR "birth dose*"[Text Word] OR "birth dose*"[Text Word] OR "birthdose*"[Text Word] OR "neonatal vaccin*"[Text Word] OR "mobile vaccin*"[Text Word] OR "outreach vaccin*"[Text Word] OR "community vaccin*"[Text Word])

AND

Context

("developing countries"[MeSH Terms] OR "global health"[MeSH Terms] OR "africa"[MeSH Terms] OR "africa south of the sahara"[MeSH Terms] OR "rural health services"[MeSH Terms] OR ("rural population"[MeSH Terms] OR "low and middle income countries"[Text Word] OR "lmic*"[Text Word] OR "africa*"[Text Word] OR "resource-limited"[Text Word] OR "resource-limited"[Text Word] OR "resource constrained"[Text Word] OR "remote"[Text Word] OR "hard-to-reach"[Text Word] OR "hard-to-reach"[Text Word])

AND

Specific vaccines

("hepatitis b vaccines"[MeSH Terms] OR "bcg vaccine"[MeSH Terms] OR "poliovirus vaccine, oral"[MeSH Terms] OR "HepB"[Text Word] OR "BCG"[Text Word] OR "OPV"[Text Word])

AND

Date range

2005/01/01:3000/12/12[Date - Publication]

Table S4. Summary of targeted website searches and gray literature sources reviewed

Platform	# Sources Reviewed	# Sources Included	Document Types Reviewed	Included Sources
Gavi, the Vaccine Alliance	2	0	News articles	NA
World Health Organization	7	2	Program reports<br>Policy briefs<br>SAGE meeting notes<br>Reviews<br>Peer-reviewed articles<br>Guidance documents<br>Training packages	Preventing Perinatal Hepatitis B Virus Transmission: A Guide for Introducing Hepatitis B Birth Dose Vaccination<br><br>Training Package for Introducing and Strengthening Hepatitis B Birth Dose
Coalition for Global Hepatitis Elimination	8	2	Peer-reviewed article	Improving hepatitis B birth dose coverage through village health volunteer training and pregnant women education<br><br>Evaluation of storing hepatitis B vaccine outside the cold chain in the Solomon Islands: Identifying opportunities and barriers to implementation
Technet-21	8	4	Checklist and readiness assessment tool, peer-reviewed article, conference presentations	Strengthening birth dose vaccination platforms and improving the quality of services that mothers and children receive in Madagascar: A mixed-methods study<br><br>Lessons learned from developing, testing and scaling innovative interventions to strengthen the birth platform in Cameroon and Nigeria<br><br>Assessment of the hepatitis B birth dose vaccination program, Papua New Guinea, 2014<br><br>Hepatitis B vaccine stored outside the cold chain setting: a pilot study in rural Lao PDR
Boost Community	1	0	Brief	NA
Project collaborators	2	2	Project documents	Introduction and Scaling of Hepatitis B Birth Dose in Uganda<br><br>Final Report, Post Pilot Introduction (PIE), of Hepatitis B Birth Dose Vaccine in the Pilot Project Sites of Ethiopia
**Total**	**28***	**10**		

*Of the 31 sources reviewed through targeted website searches and project collaborators, 5 were identified as duplicates of records already retrieved from the PubMed database search and were removed prior to screening, resulting in 28 sources reviewed.

Table S5. Data extraction form

Domain	Data element	Description
**General study information**	Lead author	*Study author name*
	Title	*Study title*
	Publication year	*Study publication year*
	Study setting: Country	*Drop-down options:*<br>• *Ethiopia*<br>• *The Gambia*<br>• *Nigeria*<br>• *Uganda*<br>• *Other: specify*
	Study design	*Drop-down options:*<br>• *Research study (peer-reviewed)*<br>• *Program report (non-peer-reviewed)*<br>• *Policy document/guidance*<br>• *Other: specify*
	Study aim	*Summary of study aims/purpose*
	Start date	*Start year*
	End date	*End year*
**Population**	Population description	*Describe the target population of the vaccine delivery strategy*
	Total number of participants	*List the total study participants*
	Location of birth	*Drop-down options:*<br>• *Out-of-facility*<br>• *Both in- and out-of-facility*<br>• *Not specified*<br>• *Other: specify*
**Vaccine**	Vaccine(s) delivered	*Drop-down options (can select multiple):*<br>• *Hepatitis B birth dose*<br>• *OPV birth dose*<br>• *BCG*<br>• *Other: specify*
	Vaccine presentation	*Drop-down options:*<br>• *Single-dose vial*<br>• *Multidose vial*<br>• *Not specified*<br>• *Other: specify*
**Delivery strategy information**	Delivery strategy description	*Describe the components involved in reaching the target population and administering the vaccine.*
	Delivery strategy type	*Drop-down options (can select multiple):*<br>• *Outreach*<br>• *Postnatal home visit*<br>• *Integration with maternal/newborn care*<br>• *Incentives*<br>• *Digital tracking*<br>• *Community tracking*<br>• *Other: specify*

Domain	Data element	Description
	Strategy implementation lessons	*Describe delivery strategy success factors, challenges, learnings*
	Strategy adaptations	*Describe how the delivery strategy is different from other strategies and/or what adaptations were made to the status quo*
	Where was the vaccine administered?	*Drop-down options:*<br>• *Facility (including health post)*<br>• *Community (including home)*<br>• *Other: specify*
	Health workers involved	*Drop-down options (can select multiple):*<br>• *Facility health worker (nurse, physician)*<br>• *Community health worker (HEW, VHT, etc.)*<br>• *Midwife / traditional birth attendant*<br>• *Other specialized health worker (pharmacist, laboratory personnel)*<br>• *Health manager*<br>• *Not specified*<br>• *Other: specify*
	Who was the vaccinator	*Drop-down options (can select multiple):*<br>• *Facility health worker (nurse, physician)*<br>• *Community health worker (HEW, VHT, etc.)*<br>• *Midwife / traditional birth attendant*<br>• *Not specified*<br>• *Other: specify*
	Health worker lessons	*Describe success factors, challenges, learnings related to the health workers involved in vaccine delivery*
	Vaccine handling	*Capture Information about vaccine handling, such as: How are vaccines stored (e.g., vaccine carriers)? Is coolant used (e.g., ice packs, cold water packs)? What is the time frame vaccines are OCC (if applicable)? Are temperature monitoring tools mentioned, including a vaccine vial monitor?*
	Cold chain used	*Drop-down options:*<br>• *Active cold chain (fridges)*<br>• *Passive cold chain (ice packs)*<br>• *Hybrid cold chain (insulated containers with temperature monitoring)*<br>• *Controlled Temperature Chain (CTC)*<br>• *Out of Cold Chain (OCC)*<br>• *Not specified*<br>• *Other: specify*
	Cold chain lessons	*Describe success factors, challenges, learnings related to the cold chain involved in vaccine delivery*
**Comparison**	Status quo	*Describe alternative strategies / status quo (e.g., facility-based immunization, no intervention)*
**Outcomes**	Outcomes of strategy	*Summarize the outcomes of the vaccine delivery strategy*
	Type of outcome reported	*Drop-down options (can select multiple):*<br>• *Vaccine coverage*<br>• *Timeliness of vaccination*<br>• *Equity*

Domain	Data element	Description
		• *Health impact (morbidity, mortality)*<br>• *Cost or cost-effectiveness*<br>• *Process improvement*<br>• *Community engagement / buy-in*<br>• *Other: specify*

Abbreviations: BCG, Bacillus Calmette–Guérin; CTC, controlled temperature chain; HBV, hepatitis B virus; OCC, out of cold chain; OPV0, oral polio vaccine birth dose; PNC, postnatal care; TBA, traditional birth attendant.
